# Vertebral Tongue-Like Deformity in Mucopolysaccharidosis VI

**DOI:** 10.5334/jbsr.2611

**Published:** 2021-09-27

**Authors:** Michiel Landen, François Eyskens, Filip Vanhoenacker

**Affiliations:** 1 UZ Leuven, BE; 2 Antwerp University Hospital, BE; 3 AZ Sint-Maarten Mechelen, BE

**Keywords:** mucopolysaccharidosis, gibbus, thoracolumbar hyperkyphosis, tongue-like vertebrae, bullet-shaped vertebrae

## Abstract

**Teaching point:** Defective development of the anterior portion of the vertebral body at the thoracolumbar junction may be an important imaging clue in the diagnosis of mucopolysaccharidosis.

## Case

**Figure 1:**
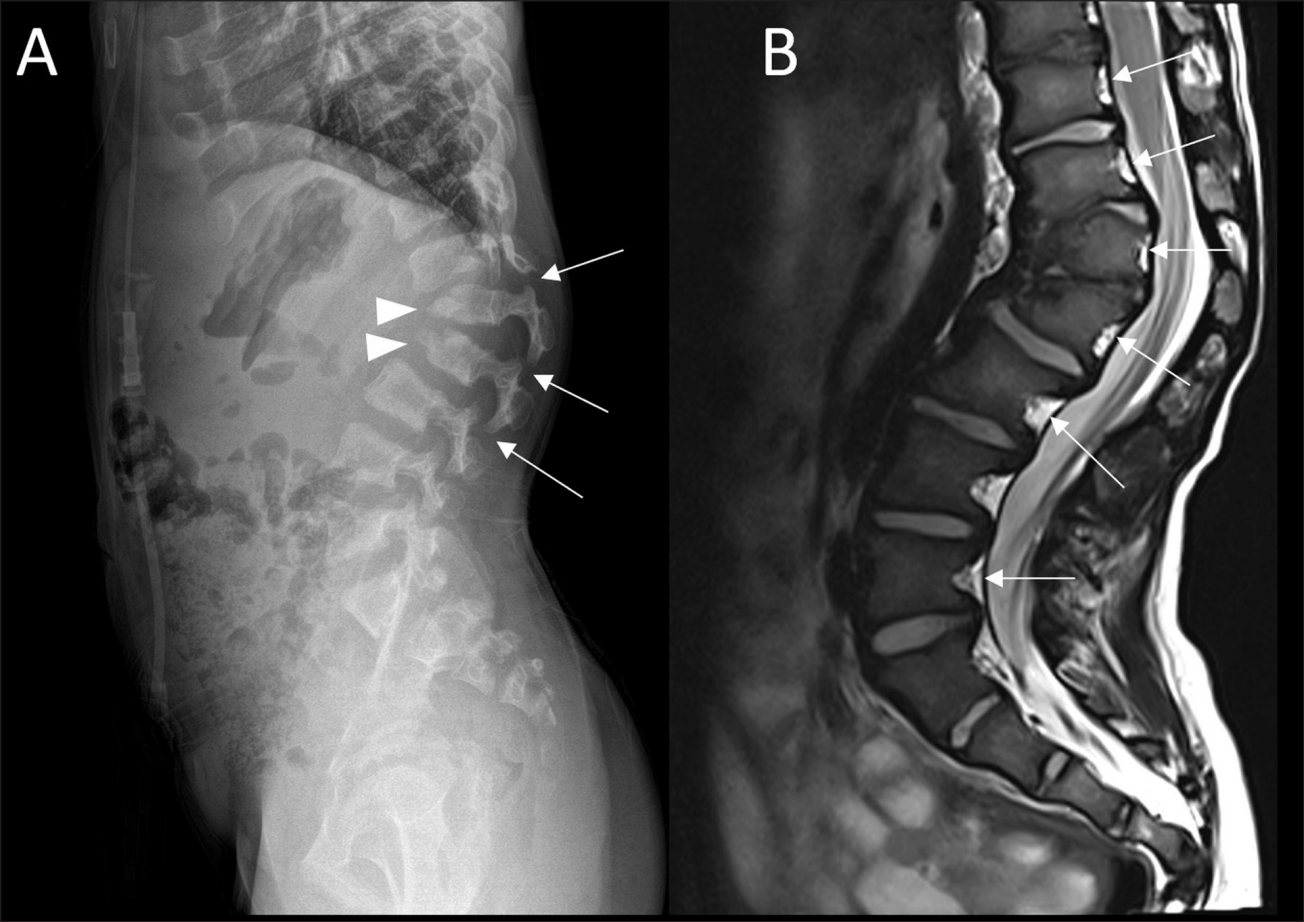


**Figure 2:**
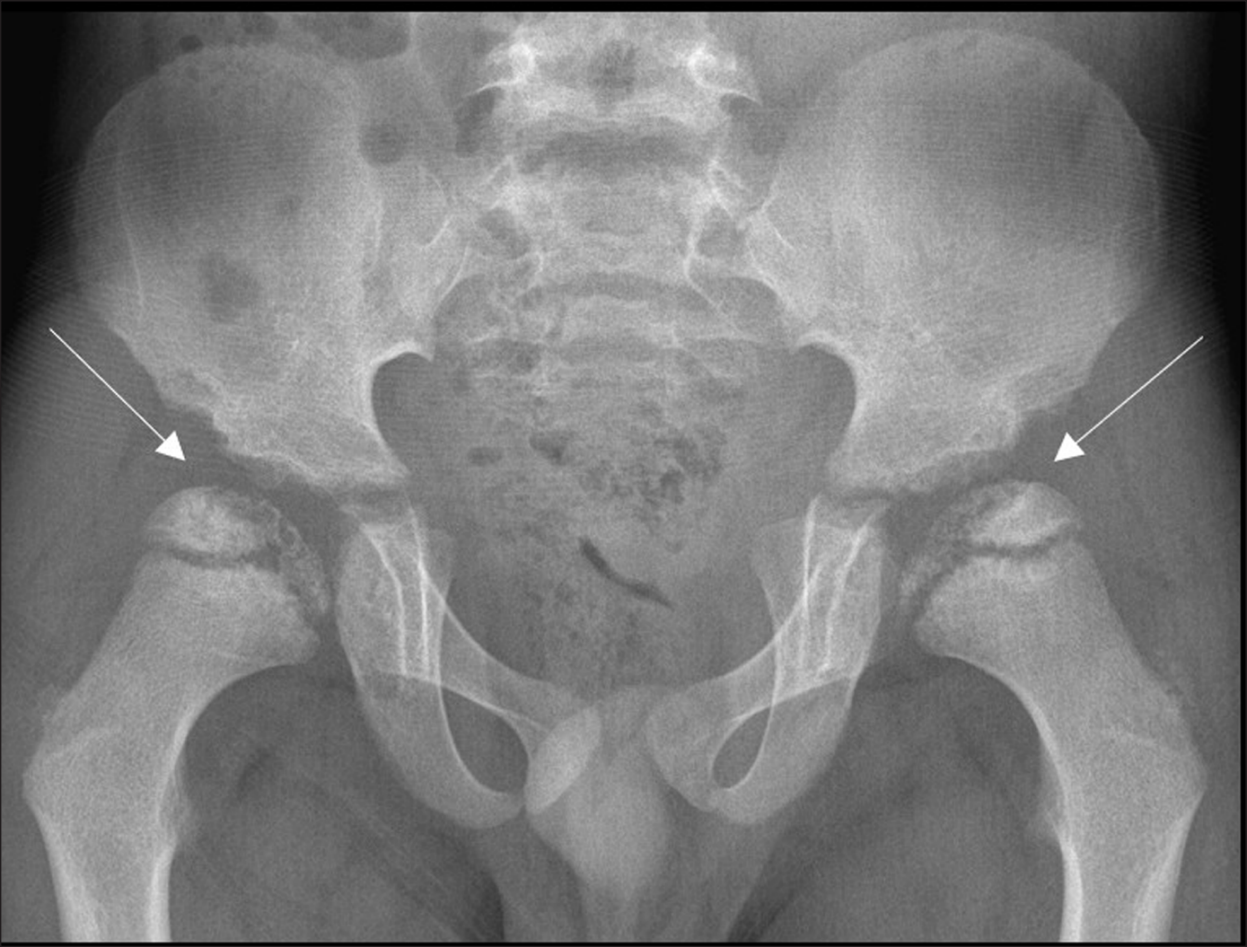


A six-year-old male presented with thoracolumbar gibbus. A full spine radiograph revealed an abnormal morphology of the vertebrae at the thoracolumbar junction, most prominent L1 and L2, associated with hyperkyphosis at the thoracolumbar junction, facet dislocation at Th12-L1, L1-L2 and L2-L3 (***Figure 1A***, white arrows) and spondylolisthesis of Th12 and L2. Anterior beaking of the vertebral bodies L1 and L2 was seen (***Figure 1A***, arrowheads), resembling a tongue or bullet. A pelvic radiograph as part of the full spine showed bilateral hip dysplasia with underdevelopment of the acetabuli and femoral epiphyses (***Figure 2***, white arrows). Medical history revealed recurrent otitis media, upper airway infections, and mild obstructive sleep apnea.

**Figure 3:**
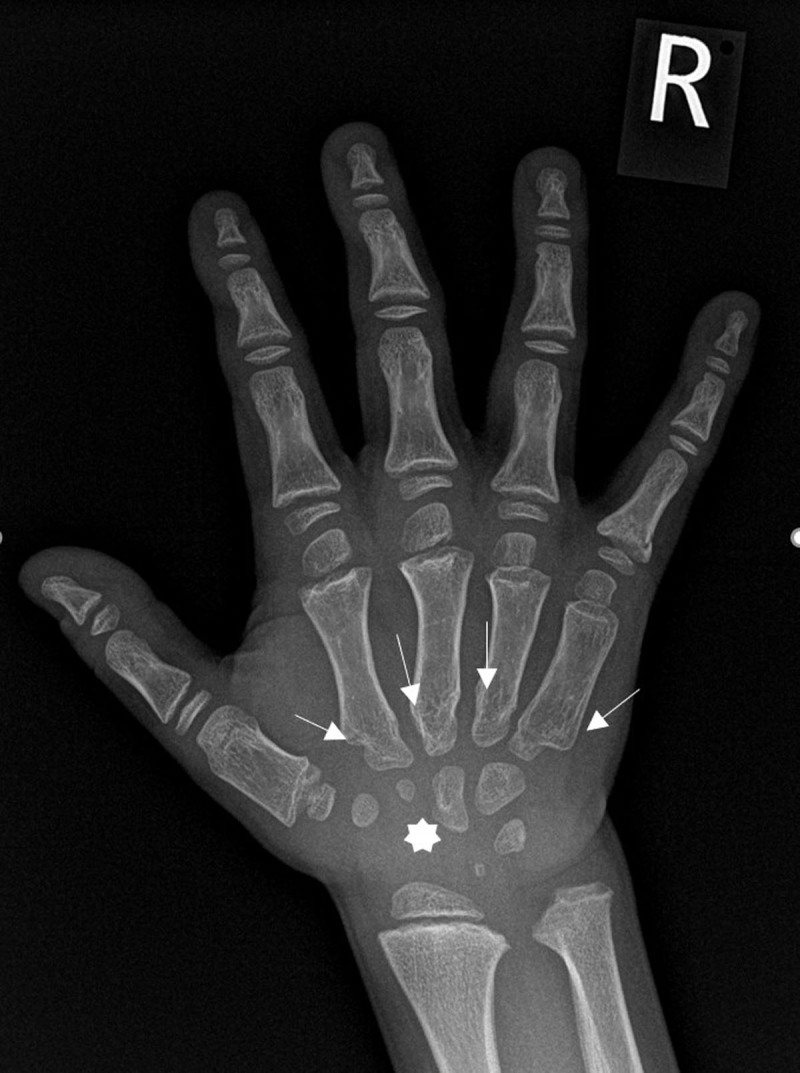


Based on the combination of clinical and imaging features a storage disease was suspected and additional examinations were performed. A radiograph of the hand showed a V-shaped deformity of the wrist, hypoplastic morphology of the proximal metacarpals (***Figure 3***, white arrows) and a delay in carpal ossification (***Figure 3***, asterix). The skeletal manifestations were suggestive of dysostosis multiplex congenita. MRI of the spine showed no spinal stenosis and no cord anomaly and confirmed the vertebrae hypoplasia with posterior scalloping (***Figure 1B***, white arrows). Urinary examination exposed high dermatan sulphate and chondroitin sulphate levels in keeping with mucopolysaccharidosis VI. The diagnosis of mucopolysaccharidosis VI was confirmed by enzymatic analysis of arylsulphatase B in leucocytes and homozygosity of the c.937C>G mutation (exon 5 ARSB gene). Both parents were carriers of the same mutation although consanguinity was not known.

## Comment

Mucopolysaccharidosis (MPS) is a lysosomal storage disorder affecting multiple organ systems. To date, seven different types of MPS are clinically identified with a varying degree of skeletal involvement between them. The musculoskeletal manifestations, designated as dysostosis multiplex congenita, are often pronounced and occur usually early in the onset. They can be recognized on radiographs even before the clinical diagnosis is made [1].

Thoracolumbar kyphosis or gibbus deformity occur in nearly all children with MPS and is secondary to hypoplastic vertebrae. Poor bone growth predominantly involves the anterior part of the vertebral bodies resulting in tongue-like or bullet-shaped vertebrae. Other manifestations of aberrant cartilage and bone growth related to MPS are macrocephaly with a thickened skull and J-shaped sella turcica, odontoid hypoplasia, short wide clavicles, paddle-shaped ribs, hip dysplasia, coxa valga, shortened long bones with cortical thinning and hypoplastic epiphyses, and bullet-shaped phalanges [1].

In conclusion, underdevelopment of the anterior vertebral bodies at the thoracolumbar junction warrants further work-up to exclude storage disease.
